# Biofeedback Posture Training for Adolescents with Mild Scoliosis

**DOI:** 10.1155/2022/5918698

**Published:** 2022-01-31

**Authors:** Mei-chun Cheung, Joanne Yip, Janelle S. K. Lai

**Affiliations:** ^1^Department of Social Work, The Chinese University of Hong Kong, Shatin, New Territories, Hong Kong SAR, China; ^2^Institute of Textiles and Clothing, The Hong Kong Polytechnic University, Hung Hom, Kowloon, Hong Kong SAR, China

## Abstract

Adolescent idiopathic scoliosis (AIS) is characterized by uneven shoulders, spinal curvature, and uneven hips, and asymmetry in paraspinal muscle activities is common in AIS. This pilot study was aimed at examining the use of a surface electromyography (sEMG) biofeedback posture training program in adolescents with mild scoliosis (Cobb′s angle < 30°) to attenuate asymmetry in paraspinal muscle activities and control the curve progression. Seven female adolescents (age, 12–14 years) with mild scoliosis (Cobb′s angle < 30°) were recruited. The participants received 30 tailor-made sessions of sEMG biofeedback posture training at a rate of one to two sessions per week for approximately 6 months. The activities of the paraspinal muscles (the trapezius, latissimus dorsi, thoracic erector spinae, and lumbar erector spinae) measured by sEMG during habitual sitting postures and spinal deformity evaluated by 3D ultrasound imaging were compared before and after training. The mean values of the root-mean-square sEMG ratio, an index of symmetry in paraspinal muscle activities of the muscle pairs between the concave and convex sides of the spinal curve, revealed significant asymmetry over the trapezius and lumbar erector spinae before the training (*p* <0.05). After the training, all seven adolescents achieved relatively more symmetrical paraspinal muscle activities over these two muscle pairs (*p* < 0.05). In two adolescents, the spinal curvature decreased by 5.7° and 5.6°, respectively, whereas the remaining adolescents showed a minimal curve progression with changes in the spinal curvature controlled under 5°. To conclude, sEMG biofeedback posture training can reduce asymmetry in paraspinal muscle activities and control curve progression in adolescents with mild scoliosis and can potentially be considered an alternative early intervention for muscle reeducation in this cohort.

## 1. Introduction

Poor torso posture is characterized by a prolonged deviation from a “neutral spine” [1]. A cohort study of the Vilnius preschool population reported that 46.9% of the 791 children in their study had trunk asymmetry caused by poor posture [2]. Longitudinal studies showed that trunk asymmetry and poor posture in prepubertal children may predict scoliosis [3] characterized by uneven shoulders, spinal curvature, and uneven hips. In addition, asymmetry in paraspinal muscle activities is observed in adolescent idiopathic scoliosis (AIS) [4–8]. Specifically, the angle of spinal curvature significantly correlated with the surface electromyography (sEMG) amplitude of the erector spinae in young school children [9]. Kwok et al. [7] compared paraspinal muscle activities between female adolescents with and without mild scoliosis (i.e., spinal curve angle less than 20°) by sEMG during habitual standing and sitting postures and found that those with thoracic- or thoracolumbar-type scoliosis had higher average root-mean-square (RMS) sEMG values on the convex side of the affected muscle regions. They showed evident asymmetry in muscle activities between the left and right upper trapezius and latissimus dorsi in the habitual sitting posture [7]. Greater sEMG values on the convex sides were significantly associated with curve progression for adolescents with AIS [10]. While most investigators concede that abnormalities in paraspinal muscle activities are likely to be secondary to spinal deformity in AIS [11], some studies suggest that asymmetry in paraspinal muscle activities may be of value for predicting curve progression [10, 12]. Therefore, posture training to improve the symmetry in paraspinal muscle activities in AIS might reduce the risk of curve progression.

The earliest postural training device for AIS was developed by Dworkin [13]. It was a small microprocessor-based postural training device that provided continuous information to patients about their posture through an audio feedback mechanism [14]. Wong et al. [15] studied the effectiveness of this device for posture training in AIS with Cobb's angles between 25° and 35°. The device achieved a curve-control success rate of 69% [15]. However, one disadvantage of this device is that patients may not hear the auditory feedback if the level of ambient noise is high. Although the device is portable, it needs to be worn for 23 hours a day for a minimum of 18 months [15], which causes discomfort and thus results in poor compliance. Later, to monitor the trunk posture, Wong and Wong [16] introduced a smart garment with integrated accelerometers and gyroscopes, which can detect postural changes in terms of the curvature variation of the spine in the sagittal and coronal planes.

Presently, the treatment options for adolescents with Cobb's angles from 10° to 25° generally involve follow-up through clinical monitoring and reexamination of the spinal curvature every 6 months. However, during the period from 10 to 16 years of age, adolescents experience a growth spurt and have a high risk of curve progression [17]. Therefore, early intervention during this stage is crucial as it may reduce the risk of curve progression [7, 18, 19]. Consequently, the use of physiotherapeutic scoliosis-specific exercise (PSSE) alone (for adolescents with Cobb′s angle < 25°) or in conjunction with braces when these are indicated (for adolescents with Cobb's angles from 20° to 45°) is recommended for adolescents with Cobb's angles between 10° and 45° [20, 21]. By incorporating personalized curve-specific exercise protocols, PSSE is aimed at reducing spinal deformity, controlling curve progression, and stabilizing improvements [22]. According to the guidelines published by the International Society on Scoliosis Orthopedic Rehabilitation Treatment (SOSORT), the standard features of PSSE are three-dimensional self-correction, training activities for daily living, and stabilization of the corrected posture through active participation [22–24]. Self-correction and stabilizing exercises, which include balance, neuromotor control, and proprioceptive training [25], are taught to each adolescent individually to improve their self-awareness of spinal deformity [22]. While a Cochrane Review found only low-quality evidence on the effectiveness of scoliosis-specific exercises for AIS [26], another systematic review showed that the majority of included studies supported the effectiveness of PSSE in reducing the curve progression rate (mainly in early puberty), and/or improving the Cobb angles (around the end of growth), and reducing brace prescription [27]. Some studies also showed the efficacy of PSSE in reducing trunk asymmetry and improving muscular imbalance [21, 28–32]. Furthermore, the results of some randomized controlled studies of PSSE supported the positive effects of PSSE on the Cobb angle outcome, appearance perception, quality of life, and back muscle endurance [33–38].

Biofeedback is a noninvasive means to improve their health and/or performance by monitoring physiological signals from their own bodies, such as their heart rate and muscle activities, and has been successfully used to treat various disorders, such as headaches and pain, and improve gait symmetry [39]. Similar to PSSE, biofeedback facilitates active participation of the users and increases their awareness of self-induced changes during musculoskeletal therapy and neuromuscular reeducation [34]. Specifically, sEMG biofeedback training is a self-controlled training of muscle activity, based on a constant biofeedback of sEMG signals recorded from a certain muscle, with the goal of muscle modification. This training has been used in clinical and research applications [39] to achieve muscle relaxation [40, 41] or reduce muscle tension in adults [42]. As an alternative or adjunctive intervention delivered alone or in combination with physiotherapy and/or occupational therapy, biofeedback has been used for neuromuscular rehabilitation and reeducation in children with brain injury, stroke, or neurological disease, such as cerebral palsy and epilepsy, and the effectiveness of its clinical application has been documented [43]. However, to date, the use of biofeedback training for muscle reeducation in adolescents with mild scoliosis has not been examined. This pilot study was aimed at evaluating the use of a 30-session sEMG biofeedback posture training program to attenuate asymmetry in paraspinal muscle activities and controlling curve progression in female adolescents with mild scoliosis, given that scoliosis progresses more rapidly in girls than in boys [44]. We hypothesized that adolescents would achieve both relative symmetry in paraspinal muscle activities after the training, compared with the activities at baseline, and controlled curve progression.

## 2. Materials and Methods

### 2.1. Participants

Seven female adolescents aged between 12 and 14 years were recruited through a school screening program during which the angle of trunk rotation (ATR) in the thoracic and lumbar regions was assessed by a scoliometer (OSI, Orthopaedic Systems Inc., Hayward, CA, USA) during the Adam's forward bend test [45]. Given that the positive predictive value of ATR ≥ 3° was reported to be 0.717 during school screening in Hong Kong [46], the spinal condition of adolescents who were suspected of having early signs of scoliosis (with ATR ≥ 3°) was further evaluated by 3D ultrasound imaging using Scolioscan™ (Telefield Medical Imaging Limited, Hong Kong) to measure the spinal deformity, which was evaluated again after completing the training. The 3D ultrasound imaging system demonstrated moderate to strong correlations (*R*^2^ > 0.72) with Cobb's angles for both the thoracic and lumber regions obtained by X-ray imaging and a very good intra- and interrater reliability with ICC larger than 0.94 and 0.88, respectively [47]. Female adolescents were excluded from the study if they had a history of surgical or orthotic treatment for AIS. The curve type was based on the Lenke classification system [48]. The demographic data of the seven recruited female adolescents are provided in Table 1. The research was conducted in compliance with the requirements stated in the Declaration of Helsinki, and the research protocol was approved by the Human Subjects Ethics Subcommittee of the Hong Kong Polytechnic University. All adolescents participated voluntarily, and informed assent from the adolescents and written informed consent for participation from their parents were solicited.

### 2.2. sEMG Measurement before and after Training

The parameters of the sEMG values were formulated based on the standards for the Surface EMG for Noninvasive Assessment of Muscles (SENIAM) [49]. The paraspinal muscles' activities were measured in terms of sEMG activities using a preamplified sensor, MyoScan (model T9503M), and a data acquisition system, FlexComp Infiniti (model T7555M; both acquired from Thought Technology Ltd., Montreal, Canada), within 1 week before starting the posture training and after completing the training. Briefly, the sEMG electrodes with ground reference from the same company (Triode T3402M) were placed onto the paraspinal muscles, namely, the trapezius, latissimus dorsi, thoracic erector spinae, and lumbar erector spinae, in pairs to determine the muscle activities along the spine in habitual sitting postures. The skin was shaved and cleaned with alcohol wipe as suggested by the SENIAM standards to reduce the impedance to less than 5 k*Ω* [49] being verified by an impedance test built in the BioGraph Infiniti software (Thought Technology Ltd., Montreal, Canada). The sEMG signals were sampled at a rate of 2048 Hz with a 10–500 Hz band pass filter and a 60 Hz notch filter to eliminate artefacts and noise. During the baseline measurement, the participants were required to sit for 3 minutes in a relaxed state to measure their muscle activities in terms of sEMG signals. The measurements were taken three times for 3 minutes each. The RMS sEMG values of the trapezius, latissimus dorsi, thoracic erector spinae, and lumbar erector spinae for each trial were calculated using BioGraph Infiniti software, and the scores for three trials of the trapezius, latissimus dorsi, thoracic erector spinae, and lumbar erector spinae, respectively, were averaged. The RMS sEMG ratio for each tested muscle pair of each participant was then calculated using the following equation [13]:

(1) $$\begin{array}{c} RMS\;sEMG\;Ratio=\frac{RMS\;sEMG\;\left(convex\right)}{RMS\;sEMG\;\left(concave\right)}. \end{array}$$

The RMS sEMG ratio is an index of symmetry in the activities of the tested paraspinal muscle pairs [7]. If the RMS sEMG ratio of one tested muscle pair is less than 1, the concave side of the muscle has relatively greater sEMG activity than the convex side. If the ratio is larger than 1, the concave side of the muscle has relatively weaker sEMG activity than the convex side. Therefore, a ratio of 1 indicates that the tested muscle pair has relative symmetry in sEMG activity between the concave and convex sides. The training was considered to be beneficial if the RMS sEMG ratio of the tested muscle pair was closer to 1 and significantly different from that at baseline.

### 2.3. Biofeedback Posture Training

An sEMG biofeedback posture training protocol was delivered using Thought Technology BioGraph Infiniti software (Montreal, Canada). Each adolescent received 30 sessions of training delivered in one to two sessions per week for approximately 6 months. During each training session, the adolescents underwent a 10-minute EMG setup procedure with an impedance check, which was followed by the baseline measurement. During posture training, they were instructed to sit in an ideal recommended posture, maintain this posture, and relax four pairs of paraspinal muscles, namely, the trapezius, latissimus dorsi, thoracic erector spinae, and lumbar erector spinae, for 5 minutes. Animated indicators on the biofeedback screen appeared whenever the sEMG values of the paraspinal muscles and the RMS sEMG ratio of the paraspinal muscle pairs fell below the threshold of specific individualized requirements. These indicators were aimed at reducing the RMS sEMG ratio between the left and right sides of the four pairs of muscles toward 1 and the individual sEMG values of each of those muscles to less than 5 *μ*V. As 12 indexes (4 ratios and 8 individual values) were required to be measured to activate the animated indicators, the exact threshold was individually tailored for an overall success rate of approximately 80%, which also motivated the adolescents to engage in the training. Therefore, animated indicators reacted in proportion to the individual sEMG values of the paraspinal muscles and the RMS sEMG ratios of the four paraspinal muscle pairs. Increased relaxation in the paraspinal muscles and symmetry in the sEMG activities over the four paraspinal muscle pairs led to an increase in the appearance frequencies of animated indicators. This posture training routine was administered five times in each session, with a 2-minute resting period between each time. Each training session lasted approximately 60 minutes.

### 2.4. Statistics

All statistical analyses were performed using the SPSS Statistic Version 25 program for Windows (IBM Corp., Armonk, NY). The RMS sEMG ratios of the four paraspinal muscle pairs were subjected to one-sample *t*-tests to identify any asymmetry (deviation from the test value of 1) in sEMG activities over the trapezius, latissimus dorsi, thoracic erector spinae, and lumbar erector spinae, before and after the training. Given the small sample size, nonparametric one-sample Wilcoxon signed rank test was also performed. A repeated measure analysis of variance (ANOVA) was performed to compare the RMS sEMG ratios of the four paraspinal muscle pairs before and after the training, followed by a post hoc pairwise comparison if the multivariate results were significant. Nonparametric Wilcoxon signed rank test was also done to compare the difference in view of the small sample size. The level of significance was set at *p* < 0.05. The difference in the degree of the spinal curve angle from baseline to posttraining was computed.

## 3. Results

**Figure 1:**
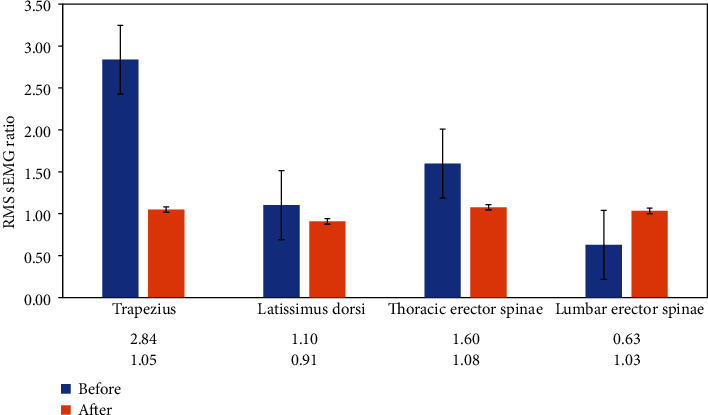
The RMS sEMG ratios of the participants before and after the biofeedback posture training. An RMS sEMG ratio of 1 indicates symmetric sEMG activity over the muscle pair. RMS sEMG refers to root-mean-square surface electromyography.

The RMS sEMG ratios of the trapezius, latissimus dorsi, thoracic erector spinae, and lumbar erector spinae muscle pairs before and after the biofeedback posture training are provided in Figure 1. Spinal deformity was evaluated using Scolioscan™, and the curve angles over the thoracic and lumbar regions before and after the training are presented in Table 1.

At baseline, the results of one-sample *t*-test showed that the RMS sEMG ratios was significantly deviated from the ratio of 1 over the muscle pairs of the trapezius (*M* = 2.837, *t*(6) = 2.800, *p* < 0.05) and lumbar erector spinae (*M* = 0.629, *t*(6) = −3.894, *p* < 0.01), suggesting a relative asymmetry of the paraspinal muscle activities in these two muscle pairs among seven participants. The results of nonparametric one-sample Wilcoxon signed rank test also showed similar significant difference for the trapezius (*p* < 0.05) and lumbar erector spinae (*p* < 0.05).

A time (pre and post) × muscle pair (trapezius, latissimus dorsi, thoracic erector spinae, and lumbar erector spinae) repeated measure ANOVA was conducted to compare the relative symmetry in sEMG activities over the four paraspinal muscle pairs before and after the training. The multivariate results showed that there was no significant difference in the main effects of time (*F*(1, 6) = 1.898, *p* > 0.05) and muscle pair (*F*(3, 4) = 5.467, *p* > 0.05), whereas the time × muscle pair interaction had significant effects (*F*(3, 4) = 6.784, *p* < 0.05, partial *η*^2^ = 0.836). A post hoc pairwise comparison revealed that the posttraining RMS sEMG ratio of the trapezius was significantly lower (*M* = 1.049, *t*(6) = 2.739, *p* < 0.05) than that at baseline (*M* = 2.837). Similarly, a significant difference was observed in the RMS sEMG ratio of the lumbar erector spinae from before (*M* = 0.629) to after (*M* = 1.033) the training (*t*(6) = −3.581, *p* < 0.05). The results of nonparametric Wilcoxon signed rank test also demonstrated similar significant difference for the trapezius (*p* < 0.05) and lumbar erector spinae (*p* < 0.05).

The results of one-sample *t*-test showed that relative symmetry in the paraspinal muscle activities was achieved over the muscle pairs of both the trapezius (*t*(6) = 0.953, *p* > 0.05) and the lumbar erector spinae pair (*t*(6) = 0.682, *p* > 0.05) after the training, i.e., their corresponding RMS sEMG ratios were closer to 1. The results of nonparametric one-sample Wilcoxon signed rank test also demonstrated similar nonsignificant difference for the trapezius (*p* > 0.05) and lumbar erector spinae (*p* > 0.05).

Spinal deformity was measured in terms of the spinal curve angle to further demonstrate the effects of the training. After the training, two participants showed a reduced curvature, with a reduction in the spinal curve angle of 5.7° and 5.6°. Though the remaining five participants exhibited a minimal curve progression over the thoracic and/or lumbar regions, the change in the spinal angle was controlled under 5° (Table 1).

## 4. Discussion

The RMS sEMG ratios shown in Figure 1 demonstrate that the seven female adolescents exhibited asymmetry in some paraspinal muscle activities before training. Specifically, the average RMS sEMG ratios of the trapezius and lumbar erector spinae muscle pairs were significantly greater or less than 1, suggesting asymmetry in these two paraspinal muscle activities before training. While the trapezius tended to show a relatively greater muscle activity on the convex side than on the concave side, the lumbar erector spinae exhibited a relatively greater muscle activity on the concave side than on the convex side. The muscle asymmetry in the trapezius was consistent with the findings of previous research on AIS patients, which revealed a higher average RMS sEMG value on the convex side of the trapezius because of lower bioelectricity activity on the concave side [7, 11]. However, a contradictory finding was reported in the lumbar erector spinae, with a higher average RMS sEMG value on the concave side than on the convex side. This difference may be attributable to the fact that our sample was quite heterogeneous in terms of the curve type based on the Lenke classification system, as shown in Table 1 and to the potential influence of the degree of spinal curvature on the sEMG values [7]. For a more conclusive implication, a larger sample that is homogeneous in terms of the curve type should be evaluated in the future to further confirm the association between spinal curvature and asymmetry in the muscle activity in terms of the RMS sEMG ratio.

After 30 sessions of biofeedback posture training, the RMS sEMG ratios of all the tested muscle regions approached 1, indicating more symmetric muscle pair activities relative to those measured before training. Significant improvement was observed in the muscle activities over the trapezius and lumbar erector spinae after training (*p* < 0.05). As the biofeedback posture training also relied on the sEMG signals as feedback for monitoring the posture, it is reasonable to speculate that the training would have significant effects on the participants' sEMG signals after training. Given that asymmetry in paraspinal muscle activity is considered as a prominent risk factor for curve progression [10, 12], another more objective outcome measure, i.e., spinal deformity, was measured before and after the training to determine the benefits of the biofeedback posture training. We speculated that reduction in asymmetry of paraspinal muscle activities may have beneficial effects in controlling the curve progression over time. Consistent with our hypothesis, after training, two adolescents (28.57%) showed an improved curve with a spinal curvature reduction by 5.7° and 5.6°, respectively, in the thoracic region, whereas the remaining adolescents (71.43%) showed a minimal curve progression with changes in the spinal curve angle controlled under 5°. The spinal curve angles did not exhibit a significant difference before and after the training, which may be due to our small sample size and that the change in muscle activity may not necessarily translate in spine curvature change. However, the results provide initial evidence that the biofeedback posture training can attenuate asymmetry in paraspinal muscle activities and control the curve progression among adolescents with mild scoliosis.

Mild scoliosis adversely affects the quality of life of adolescents in terms of lower self-image, increased back pain, and unhappiness with their lives [50, 51]. Presently, the treatment options for adolescents with spinal curves from 10° to 25° are generally followed up through clinical monitoring and reexamination of the spinal curvature every 6 months. Although spinal curves in a majority of adolescents (approximately 80–90%) are nonprogressive, the remaining adolescents are at risk of curve progression during puberty (age 10–16 years) if they are skeletally immature. Therefore, the adverse effects of poor posture and/or spinal deformity on adolescents' health and well-being can be prominent and enduring. According to the guidelines published by SOSORT, PSSE and braces are the only treatment options supported by level 1 evidence, and PSSE alone or in conjunction with braces when these are indicated is recommended for adolescents with spinal curves from 10° to 45° [20, 21]. Given that biofeedback training shares key features with PSSE in its clinical application, including active participation and increase in self-awareness of the spinal deformity to promote self-correction [22, 52], the results of our study are consistent with those of previous studies regarding the use of PSSE in AIS, which found that active participation of AIS adolescents in an individualized selection of exercises could be regarded as a preventive measure to improve their paraspinal muscle activity [32]; reduce the curve progression, trunk asymmetry, and brace prescription [21, 27, 53]; and/or enhance their balance, strength, and mobility [21, 28–32, 54]. The findings of our study substantiate that, as a type of noninvasive intervention in which individuals are trained to improve their health and/or performance by monitoring physiological signals from their own bodies, such as muscle activities, sEMG biofeedback posture training can potentially be used as an alternative early intervention for muscle reeducation in AIS, especially among adolescents with mild scoliosis who are at a high risk of curve progression.

This pilot study explores the possibility of implementing noninvasive sEMG biofeedback posture training for adolescents with mild scoliosis. The significance of this study should be considered in light of the following limitations. First, because the sample size was limited, the difference in the spinal curve angle before and after training did not reach significance, and its association with the change in the RMS sEMG ratios could not be evaluated. Second, this study recruited adolescents with different curve types based on the Lenke classification system and severities of spinal curvatures, with Cobb's angles ranging from 7.9° to 27.6°. This heterogeneity allowed us to analyze the extent of applicability of the proposed training program to different types of scoliosis. However, the effectiveness of the biofeedback posture training on AIS with a particular curve type could not be evaluated. In addition, it is difficult to draw conclusions on its effectiveness based on the current single-arm study. Hence, further studies with a control group for comparison and a larger sample size that is homogeneous in terms of the range of Cobb's angles and curve types are recommended. Given that curve progression occurs over time, it would be conceivable to conduct a randomized controlled trial to further explore and confirm the effectiveness of such biofeedback posture training on AIS patients and its sustainability over time.

## 5. Conclusion

After receiving 30 tailor-made sessions of sEMG biofeedback posture training at a rate of one to two sessions per week for approximately 6 months, seven female adolescents (age, 12–14 years) with mild scoliosis (Cobb′s angle < 30°) demonstrated more symmetrical paraspinal muscle activity over the trapezius and lumbar erector spinae muscle pairs. In two adolescents, the spinal curvature decreased by 5.7° and 5.6°. The findings of this pilot study provide preliminary empirical evidence to support that sEMG biofeedback posture training can be used to attenuate asymmetry in paraspinal muscle activities among adolescents with mild scoliosis and can potentially be considered an alternative early intervention to improve their posture and control their curve progression.

An earlier version of this manuscript has been submitted to Research Square as a preprint in December 2020 [55].

## Figures and Tables

**Table 1:** Demographic information of the participants and their spinal deformity angles before and after the biofeedback posture training.

							Before		After		Difference	
Subject no.	Age (years)	Lenke's classification	Convex side	Height (cm)	Weight (kg)	BMI	Thoracic angle (°)	Lumbar angle (°)	Thoracic angle (°)	Lumbar angle (°)	Thoracic angle (°)	Lumbar angle (°)
A	14	Type 6	Right (thoracic), left (lumbar)	156	44.6	18.3	10.6	12.8	12	14.5	1.4	1.7
B	14	Type 6	Right (thoracic), left (lumbar)	142	39	19.3	7.9	12.1	8.2	9.8	0.3	-2.3
C	14	Type 6	Right (thoracic), left (lumbar)	152	38.5	16.7	8.3	17.4	10.2	15.1	1.9	-2.3
D	13	Type 1	Right (thoracic)	163	45.1	17	21.4	N/A	25.3	N/A	3.9	N/A
E	13	Type 5	Left (lumbar)	154	46.3	19.5	N/A	9.6	N/A	10.3	N/A	0.7
F	13	Type 1	Left (thoracic)	151	53.6	23.5	17.6	N/A	11.9	N/A	−5.7^a^	N/A
G	12	Type 3	Right (thoracic), left (lumbar)	155	40	16.5	27.6	15.4	22	15.4	−5.6^a^	0

^a^More than 5° reduction in spinal deformity. BMI: body mass index; N/A: not applicable.

## Data Availability

The data used to support the findings of this study are available from the corresponding author upon request.
